# OA25 Constitutional trisomy 8 mosaicism is associated with a paediatric incomplete Bechet’s-like phenotype

**DOI:** 10.1093/rap/rkac066.025

**Published:** 2022-09-28

**Authors:** Fahim Patel

**Affiliations:** Birmingham Children's Hospital, Birmingham, United Kingdom

## Abstract

**Introduction/Background:**

Behcet’s disease is a rare paediatric diagnosis and to date there is no single confirmatory gold-standard test or approach. Instead, decisions are clinician-led based on experience, pattern recognition and categorisation according to international criteria. Diagnostic dilemmas arise when patients present at early ages with an incomplete or atypical phenotype. Such cases should prompt clinicians to consider Bechet-like mimics with emphasis of monogenetic and chromosomal causes in younger children.

**Description/Method:**

A 5-year-old boy with constitutional Trisomy 8 mosaicism was referred to a Bechet’s disease National Centre of Excellence paediatric clinic with cyclical episodes of fever, mouth ulcers, pseudofolliculitis and headaches.

During the initial outpatient assessment period (6-12 months) symptoms were reported every 2-3 months and would last for around 1-2 weeks at a time. Physical examination and a photo diary reveal oral ulcers and rashes. He had pre-existing skeletal dysmorphism with severe pectus carinatum, scoliosis and widespread limb contractures secondary to Trisomy 8. Serial blood testing around episodes showed an associated inflammatory response.

Although his phenotype was incomplete; with evidence of recurrent autoinflammation a trial of immunosuppression with colchicine was commenced. He displayed a dose-dependent improvement as evidenced by reduction in the frequency and severity of episodes.

His Behcet’s-like disease remains well controlled and to date there has been no amyloidosis, ocular, gastrointestinal or cardiovascular complications.

**Discussion/Results:**

Behcet’s disease is a multisystem chronic vasculitis presenting with widespread mucosal involvement occurring in a relapsing-remitting fashion. Classic manifestations are recurrent sterile aphthous ulcers, genital ulcers, mucosal eye involvement and a positive Pethargy test.

It usually presents with all of these classic features by the 3rd or 4th decade of life; children can be affected but usually present with paucity of symptoms not reaching diagnostic criteria. As such, the accumulation of symptoms during a follow-up period may lead to a diagnostic lag of around 3-5 years.  The young age of this patient therefore raised clinical suspicion for an alternative diagnosis.

The aetiology of Bechet’s disease has elucidated rheumatologists since the first description in 1937. An association with HLA-B51 antigen is well documented but not fully understood. The advent of widely available next-generation genetics provides important clues to its aetiology and helps delineate monogenic and chromosomal mimics. To date 21 genetic loci have been identified through genome-wide association studies and least 11 monogenic and chromosomal mimics been described in the literature.

Constitutional Trisomy 8 mosaicism has been shown to be a chromosomal-driven mimic of Behcet disease. It is rare (1-25.000 to 1-50.000 live births) with a 5:1 male predilection; those affected present with a spectrum of abnormal facies, vertebral and limb anomalies including contractures.

Interestingly a similar Bechet-like disease is also seen in patients who acquire Trisomy 8 mosaicism in cells affected by myeloid malignancies. Such cases are more numerous and predate those associated with the constitutional form.

The final common pathogenesis is thought to be activation of the NF-κB pathway. Reports postulate overexpression of several cytokine genes, production of proinflammatory cytokines and reactive oxygen species. The exact pathogenesis is not fully understood, further research may reveal clues to understanding both Constitutional Trisomy 8 mosaicism and Behcet Disease.

**Key learning points/Conclusion:**

Learning points:

1. Consider monogenic and chromosomal Behcet Disease mimics in those patients who present at young ages or at any time with atypical or incomplete features.

2. Our patient with Constitutional Trisomy 8 Mosaicism with Behcet-like disease showed a dose-dependent improvement with colchicine.

